# Identifying FAAH Inhibitors as New Therapeutic Options for the Treatment of Chronic Pain through Drug Repurposing

**DOI:** 10.3390/ph15010038

**Published:** 2021-12-28

**Authors:** Anca Zanfirescu, Georgiana Nitulescu, Dragos Paul Mihai, George Mihai Nitulescu

**Affiliations:** Faculty of Pharmacy, “Carol Davila” University of Medicine and Pharmacy, Traian Vuia 6, 020956 Bucharest, Romania; anca.zanfirescu@umfcd.ro (A.Z.); dragos_mihai@drd.umfcd.ro (D.P.M.); george.nitulescu@umfcd.ro (G.M.N.)

**Keywords:** endocannabinoids, chronic pain, N-arachidonoylethanolamide, fatty acid amide hydrolase, drug repurposing, montelukast, repaglinide, revefenacin

## Abstract

Chronic pain determines a substantial burden on individuals, employers, healthcare systems, and society. Most of the affected patients report dissatisfaction with currently available treatments. There are only a few and poor therapeutic options—some therapeutic agents are an outgrowth of drugs targeting acute pain, while others have several serious side effects. One of the primary degradative enzymes for endocannabinoids, fatty acid amide hydrolase (FAAH) attracted attention as a significant molecular target for developing new therapies for neuropsychiatric and neurological diseases, including chronic pain. Using chemical graph mining, quantitative structure–activity relationship (QSAR) modeling, and molecular docking techniques we developed a multi-step screening protocol to identify repurposable drugs as FAAH inhibitors. After screening the DrugBank database using our protocol, 273 structures were selected, with five already approved drugs, montelukast, repaglinide, revefenacin, raloxifene, and buclizine emerging as the most promising repurposable agents for treating chronic pain. Molecular docking studies indicated that the selected compounds interact with the enzyme mostly non-covalently (except for revefenacin) through shape complementarity to the large substrate-binding pocket in the active site. A molecular dynamics simulation was employed for montelukast and revealed stable interactions with the enzyme. The biological activity of the selected compounds should be further confirmed by employing in vitro and in vivo studies.

## 1. Introduction

Endocannabinoids are lipid mediators released on demand from membrane phospholipid precursors [1]. They target the cannabinoid receptors CB1 and CB2, and several other receptors, such as GPR55 [2], peroxisome proliferator-activated receptors (PPARs) [3], and vanilloid receptors (TRPV1) [4].

The components of the endocannabinoid system play a key modulatory role in nociception [1,5]. Cannabinoid receptors and ligands are expressed ubiquitously throughout the pain processing pathways, from peripheral sites, such as peripheral nerves and immune cells, to central integration sites such as the spinal cord, and higher brain regions [1,5].

Although cannabinoid receptor agonists that act directly on CB1 receptors produce antinociceptive and anti-inflammatory effects across a wide range of preclinical models, the psychomimetic side effects they induce reduce their therapeutical potential [6,7]. Increasing the concentration of endocannabinoids, such as N-arachidonoylethanolamide (anandamide, AEA) rather than administering exogenous agonistic agents, is shown to possess various pharmacological effects without cannabinoid-like adverse events [8,9]. Increasing AEA levels by inhibiting fatty acid amide hydrolase (FAAH; EC 3.5.1.99), the main metabolic enzyme degrading this endocannabinoid, was shown to have a great variety of therapeutical applications including chronic pain, refractory anxiety and depression, multiple sclerosis, and cancer without characteristic cannabinoid intoxication symptoms, e.g., catalepsy, reduced body temperature, or stimulated feeding [10,11,12,13].

FAAH-knockout mice have enhanced levels of AEA and display a CB1 receptor-mediated hypoalgesic phenotype [14]. In humans, a microdeletion in dorsal root ganglia and brain-expressed pseudogene, FAAH-OUT, or a common functional single-nucleotide polymorphism in FAAH conferring reduced enzymatic expression and activity, lead to similar results [15].

The therapeutical potential of FAAH inhibition led to a great interest in developing more potent and selective enzymatic inhibitors. Various structure-activity relationship (SAR) studies have been performed on both irreversible [16,17] and reversible inhibitors [18], important structural features of such molecules being identified: the presence of an electrophilic carbon [11], an activating heterocycle with intrinsic electron-withdrawing properties which increases the electrophilic character of the reactive carbonyl [19]. The incorporation of additional basic nitrogen into the activating heterocycle enhances inhibition even more [19]. Most inhibitors possess a fatty acid side chain connecting two structural elements (e.g., an aryl and a heterocycle)—the length and degree of saturation of the fatty acid chain as well as π-unsaturation at the position corresponding to the oleyl alkene are important features for FAAH inhibitors and such insertion of aryl groups is characteristically found in most FAAH inhibitors, aromatic groups establishing hydrophobic interactions within the enzymatic acyl chain-binding pocket [18].

Development of reversible FAAH inhibitors and characterization of their interaction with the enzymatic substrate through molecular docking found three key enzymatic regions: the acyl-chain binding site—a highly hydrophobic membrane access channel (MAC) defined by Leu192, Phe194, Phe244, Leu380, Phe381, Leu404, Phe432, Met436, and Ile491- where hydrophobic contacts are established with a potential ligand, the catalytic triad Ser241–Ser217–Lys142 located in an oxyanion hole (defined by Ser241− Gly240−Gly239−Ile238) [20], and the hydrophilic cytosolic port [21]. The latter connects the catalytic region with the cytosolic compartment of the cell, the presence of the polar residues (Lys142 and Thr236) favoring the leaving group departure toward the cytosol after substrate hydrolysis. Other residues important for the hydrolytic activity of FAAH are Phe432 and Trp531, which act as a dynamic paddle, directing and orienting the substrate during catalysis [22].

The substances with increased inhibitory potency were further submitted to preclinical testing. Pharmacological inhibition of FAAH increased AEA concentrations in rats [23,24,25,26]. Both irreversible (e.g., URB597) and reversible (e.g., OL-135) inhibitors of FAAH produce pharmacological effects similar to those observed in FAAH −/− mice, including antinociception in various animal models of pain using thermal or chemical nociceptive stimuli [26], and consistent anti-hyperalgesic effects in carrageenan-induced inflammation [27], complete Freund’s adjuvant-induced osteoarthritis [28], as well as in models of neuropathic pain [29].

The results of the in silico and preclinical testing lead to several FAAH inhibitors entering clinical trials for the assessment of their efficacy in neuropsychiatric diseases, including pain conditions: compounds PF-04457845 [24], JNJ-42165279 [30], SSR-411298 [31], V-158866 [32,33], and URB597 [32]. Similar to preclinical observations, clinical studies showed no adverse effects commonly associated with exogenous cannabinoids, such as impairment in cognition, motor coordination, and psychoses [24,30,32]. We must highlight however that the effects of chronic treatment are still to be observed.

Compound BIA 10-2474 was also a FAAH inhibitor, given to healthy volunteers to assess safety. After the first two initial studies went smoothly, the effect of multiple doses was evaluated. The highest tested dose (50 mg) led to the development of severe adverse reactions in 6 participants—these reactions were mainly neurological, with one participant entering a coma which progressed rapidly to brain death. For the rest of the participants, clinical improvement occurred within a few days. The trial was suspended [34].

Although FAAH is an extremely attractive molecular target, assessing the global effects of new molecules is rather difficult (e.g., predicting interactions with off-targets). Drug repurposing is a strategy that allows avoiding unpredictable adverse effects, as it uses compounds that have already been tested in humans and have demonstrated an acceptable level of safety and tolerability, for a medical condition other than the one originally intended. In this way, toxicities not predicted by preclinical research can be avoided while the issue of efficacy can be thereafter evaluated by clinical trials. Our purpose was to employ in silico screening studies based on chemical graph mining, classification, and regression quantitative structure–activity relationship (QSAR) modeling, and molecular docking techniques to identify drugs already approved for other indications as non-toxic and selective FAAH inhibitors and to offer real solutions in treating chronic pain [35]. As far as we know, this is the first study that successfully identifies repurposed molecules as potential FAAH inhibitors.

## 2. Results

### 2.1. Datasets

A raw dataset of 1976 compounds was extracted from the ChEMBL database [36] consisting of chemical structures and corresponding half-maximal inhibitory concentrations (IC_50_, M) on FAAH1. The dataset was curated by removing duplicated records and imprecise biological values yielding a final working dataset of 1244 compounds noted as FI.

Using the FI set of compounds, two groups of inhibitors were selected based on their pIC_50_ (M) values. Set FI-S included the strong inhibitors having pIC_50_ values above 8 and counted for 248 structures, while set FI-W incorporated 164 weak inhibitors having their pIC_50_ values under 5. The compounds from subset FI-S were labeled as active (1) and those from subset FI-W as inactive (0).

To reduce the errors, the decoy compounds were chosen to have similar physicochemical properties as the FI group [37]. The ChEMBL database was screened for all the compounds with molecular weight (MW) in the 150–620 g/mol range, logarithm of the partition coefficient (logP) from −0.8 to 9.2, hydrogen bonds acceptors (HBA) between 1 and 12, and hydrogen bonds donors (HBD) between 0 and 4. The upper and lower thresholds for the aforementioned descriptors were the same for the FI dataset. The resulting 1,694,566 structures were randomly selected to obtain the decoy set (named here as DCY) consisting of 12,440 compounds. The set AL consists of the union of FI and DCY sets.

The 11,172 structures downloaded from DrugBank [38] were filtered using their MW, logP, HBA, and HBD values, applying the same ranges as those used for the selection of the DCY set. The organometallic compounds were eliminated from the filtered set resulting in a final database with 7166 structures. The removal of the organometallic compounds was based on the fact that no such compound was found in the active set of compounds and that such structures can lead to errors in the computation of the molecular descriptors.

### 2.2. Molecular Descriptors

Several 1873 descriptors were computed for all the compounds in the FI set. A series of 260 descriptors were removed from the analysis due to their constancy.

### 2.3. Murcko Frameworks Profile

The Murcko framework (MF) represents all the ring systems in each compound analyzed and the atoms linking them, while all the side-chain atoms are removed [39]. The FI set returned 546 MF structures, while the DCY set yielded 10,466 structures. From the group of 546 MF structures, 520 were generated only by the compounds in the FI set. These scaffolds (MF01-520) are formed by 1076 (86.5%) of the compounds in the FI set. Only 26 structures were common to both the FI set and DCY group.

The size of the side-chain elements was calculated as the difference between the molecular weights of each compound and its corresponding MF structure. The obtained value was named SC. For the FI set, the SC value ranged between 0 and 376.54 with an average of 70.74, while in the DCY set the range was from 0 to 531.80 and the average was 93.42. The side-chain elements contain between 0 and 27 heavy atoms in the FI set and between 0 and 35 in the DCY set.

A total of 191 compounds from the FI set (15.35%) have no side-chain elements in their structure (SC = 0), while in the DCY set the number is 706 compounds, representing a significantly lower percentage of 5.68%. The number of compounds having as side-chain elements only halogen atoms is 236 from the FI set (18.97%) and 648 for the DCY set (5.21%). The substitution with at least one –OH group is present in 93 compounds from the FI set (7.48%) and in 2398 compounds (19.28%) from the DCY set. The range of the number of major simple substitution groups found in each set are presented in Table 1.

### 2.4. Bemis-Murcko Skeletons Profile

Bemis-Murcko (BM) skeletons represent the molecular frameworks resulting after the removal of the side-chain atoms and atom labels and the conversion of all bond types to single ones [39]. The transformation of all the 1244 structures in the FI dataset resulted in 287 distinct BM skeletons. Several compounds (48, 3.86%) contain no ring structures, thus generating no scaffold. The distribution of the frequency of occurrence for each scaffold follows the power law (R^2^ = 0.942).

The 12,440 compounds from the DCY set generated 5235 unique BM structures. The null skeleton was generated by 57 compounds, representing 0.46% of the set. The number of compounds that generate each individual BM skeleton was counted for both FI and DCY sets. The ratio of these two counts divided by 0.09091 was used as a performance score (P-BM) of the skeleton’s importance in generating FAAH inhibitors. A value of 1 is equivalent to the random probability (0.09091) of finding a FAAH inhibitor in the AL set. A statistically significant value above 1 indicates the potential of that BM skeleton to generate FAAH inhibitors.

**Figure 1:**
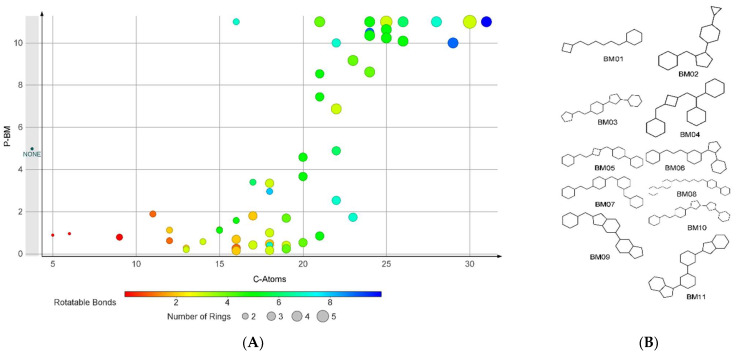
The analysis on BM skeletons: (**A**) The relation of P-BM value and number of carbon atoms, number of rings, and number of rotatable bonds for the BM skeletons with non-zero values; (**B**) the chemical structures of the BM skeletons generated by compounds found only in the FI set.

The analysis indicated 35 skeletons (BM01–BM35) with P-BM values significantly over 1. The null BM skeleton (BM36), corresponding to compounds that lack a ring structure, is associated with a P-BM value of 4.98. The scaffolds BM01–BM35 contain between 2 and 5 ring structures linked by several bonds varying in the range of 1 to 10. For most of the fragments, the P-BM value increases with the number of carbon atoms in their structure and the number of rotatable bonds (Figure 1).

Using the skeletons BM01–BM36 as filters to find FAAH inhibitors in FI and DCY sets has a sensitivity of 0.563 and a specificity of 0.950.

For the set of descriptors BM01–BM36 a performance score I-BM was calculated as the average value of the reported pIC_50_ values of all the compounds containing the respective skeleton. The obtained I-BM values were in the range of 4.03 to 9.15.

### 2.5. Plain Ring Analysis

The analysis of all the 13,684 structures in the AL set resulted in 2665 distinct plain rings (PR) scaffolds. The compounds from FI set generated 119 PR fragments, while the compounds in the DCY set returned 2651 scaffolds. The number of compounds that produce each individual PR scaffold was counted for both sets and the quotient of these two counts was divided by 0.09091 to obtain the PR performance score (P-PR). A value above 1 indicates the potential of that PR scaffold to produce FAAH inhibitors.

**Figure 2:**
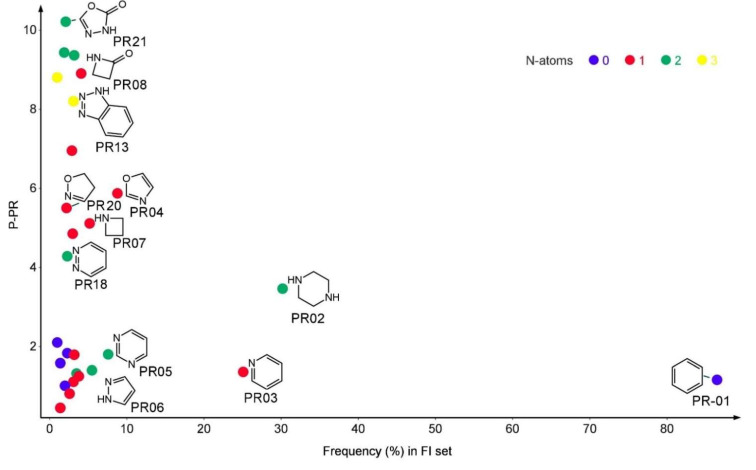
The plain rings (PR) performance score in relation to the frequency of distribution in the FI set and the number of nitrogen atoms. The plots represent only the PR structures with a prevalence of over 1%.

The most frequent PR scaffold found in the structure of FAAH inhibitors was benzene (PR1) with a frequency of 86.5%, followed by piperazine (PR02) with a frequency of 30.2%, and pyridine (PR03) found in 25.1% of compounds in FI set. The majority of the PR substructures contain nitrogen atoms, and their number was used to graphically present them. The relationship between the frequency and the P-PR score is presented in Figure 2.

A total of 81 PR scaffolds (PR01–PR81) present a P-PR value over 1. These PR structures contain between 4 and 21 non-H atoms and between 1 and 4 ring closures. The majority (91.36%) of the PR fragments incorporate at least an electronegative atom. To use the PR scaffolds to identify FAAH inhibitors, we chose as threshold the P-PR value of 3, resulting in 49 PR filtering structures. Several compounds (822) from the FI set contain at least one of the 49-PR structures, corresponding to a sensitivity of 0.661. The specificity of these filters was 0.90.

The presence of any BM01–BM36 scaffold or any of the 49-PR set of structures in the structure of a compound can be used to identify new FAAH inhibitors. Applied to the training set AL, the sensitivity of the method is 0.82 and its specificity is 0.86.

### 2.6. Classification Model

For each of the calculated 1613 descriptors, an independent sample *t*-test was performed to identify those with statistically different values between subsets FI-S and FI-W. Significant differences (confidence interval 95%) were observed for 1137 descriptors. For this array of descriptors, a receiver operating characteristics (ROC) analysis was performed. Seventy-seven descriptors returned values of the area under the curve (AUC) parameter over 0.8. The ROC curves were used to establish the cutoff values of each descriptor considering a minimum of 0.75 for both the sensitivity and specificity. This condition was obtained for only 11 descriptors, presented in Table 2. Three of the relevant descriptors are 2D, and 8 descriptors are 3D belonging to the classes of the weighted holistic invariant molecular (WHIM) descriptors, radial distribution function (RDF) descriptors, and charged partial surface area (CPSA) descriptors.

The array of values corresponding to the group of the 11 relevant descriptors were transformed in their flag type values based on their relation to the cutoff value of each descriptor. The flag values were used to perform a binary logistic regression resulting in the following equation:

(1) Log(P/1-P)=1.323×flgWPSA1+0.736×flgSpMAD_D+1.709×flgRDF85m+1.076×flgCrippenLog−2.103

where P is the probability of the compound to be active.

The regression function is based only on 4 descriptors and has a sensitivity of 0.887 and a specificity of 0.75. A positive value of the function indicates a potential FAAH inhibitor.

### 2.7. Molecular Docking

**Figure 3:**
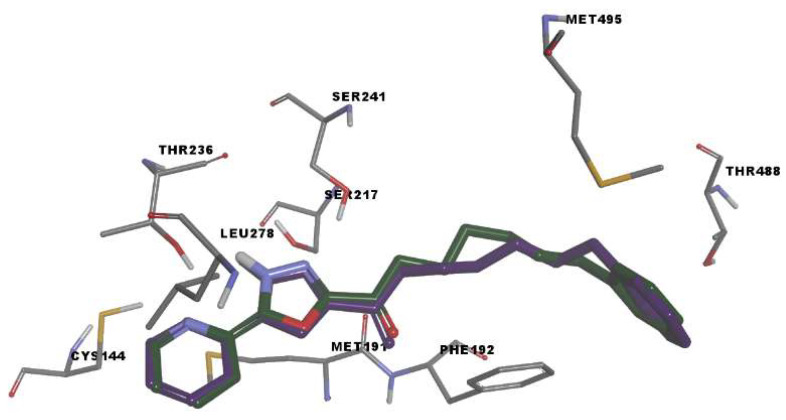
Rigid receptor docking (RRD) of the co-crystalized ligand at the active site of FAAH. It shows a cognate re-docked pose (purple) of JG1 compared with a co-crystallized pose (green).

We used molecular docking to evaluate protein-ligand interactions and to predict their binding affinity to FAAH. The molecular docking protocol was validated by docking the co-crystallized FAAH inhibitor JG1 to the target protein and calculating the root-mean-square deviation (RMSD, Å) between the experimental and predicted complexes after superposition (RMSD = 0.2345 Å; Figure 3).

The simulated protein-JG1 complex revealed that in non-covalent binding mode, the inhibitor interacts with key residues Ser217 and Ser241 and participate in halogen interactions with residues delimiting the oxyanion hole (Ile238–Ser241) while the terminal phenyl group in the acyl side chain of the inhibitor establishes weak interactions in the cytosolic port.

**Figure 4:**
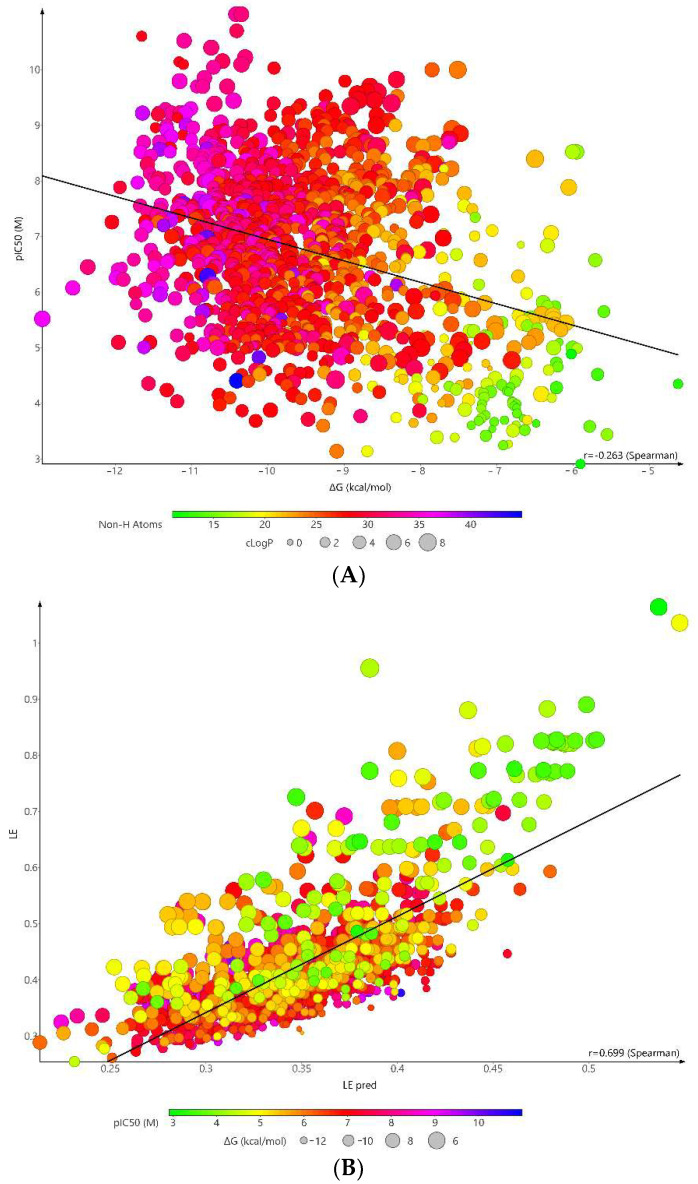
Correlation plot between experimental pIC_50_ (M) and (**A**) binding energy (kcal/mol) (R^2^ = 0.1118); (**B**) Ligand efficiency (R^2^ = 0.533) of FAAH inhibitors in molecular docking simulations.

The docking scores (ΔG) of FAAH inhibitors ranged from −12.95 to −4.63 kcal/mol with a mean value of −9.44 ± 0.13 kcal/mol. A low squared correlation coefficient between experimental pIC_50_ and the binding energy was obtained (R^2^ = 0.1118). The correlation became stronger after reporting the binding energy to the compound’s size (R^2^ = 0.533) (Figure 4). Previous studies have shown that low correlation still allows differentiating active compounds from decoys [40].

Mean ΔG values of weak (−8.12 ± 1.44 kcal/mol) and strong inhibitors (−9.71 ± 1.14 kcal/mol) were statistically different (Mann-Whitney test, *p* < 0.0001), indicating the docking algorithm allows categorization of molecules as active or inactive. Because most of the compounds with high efficacy values presents low pIC_50_ values, we chose an upper threshold of 0.4. The low threshold was set to 0.25 for the efficacy parameter, and −6 for the binding energy. A proportion of 97.18% compounds from the FI-S set fall under these filters, compared to 51.22% of the compounds from the FI-W set.

Eighty amino acids are involved in overall interactions with the compounds of the FI set, the most frequent being Phe192 (99.44%), Met495 (86.82%), The488 (84.08%), Leu404 (81.67%), and Ser193 (81.11%). On average, a compound of FI set interacts with 22.06 ± 3.77 amino acid residues (values ranging between 11 and 32), with a statistically relevant difference between the sets of strong and weak inhibitors (mean number of amino acid residues involved in interactions of 23.94 ± 3.08 vs. 19.22 ± 4.4, *p* < 0.0001, *t*-test).

### 2.8. Repurposing Study

For each compound in the AL set, a global performance score was calculated using the PR01–PR49 and BM01–BM35 scaffolds. If a compound contained several PR elements, the average of their corresponding I-PR values was calculated. The obtained value was added to the I-BM value corresponding to the compound’s BM skeleton to generate the repurposing score (RpS).

The area under the ROC curve of RpS was 0.833. Based on the ROC analysis and using a 0.9 sensitivity, the RpS threshold was set to 5.17. All the compounds with an RpS value above this value would be considered potential FAAH inhibitors. The specificity of the model is however low, with a value of 0.59. When applied to the FI-S set, the model identifies 94.78% of the compounds. The model identifies 129 compounds (78.66%) from the FI-W set. These hits are not considered false positives and this value indicates that the model identifies better the potent inhibitors, compared to the weak ones.

The DB set was filtered using the ranges for nF, nCl, nBr, and nI as presented in Table 1. The filtered set contains 7133 structures that were transformed in their corresponding BM and PR fragments. The RpS score was calculated for all the compounds. A number of 3124 structures were found to have a RpS value higher than 5.17. The compounds are classified as approved (650), experimental (1712), investigational (696), and others (66).

The values of the molecular descriptors identified by the classification analysis were calculated for all the 3124 DB compounds and filtered using the logistic regression described by Equation (1). A number of 341 DB compounds were identified to have an over 70% probability to be highly active.

A molecular docking screening was performed on all the 341 DB compounds, returning binding energies in the range of −13.033 to −6.096 and efficacy values between 0.150 and 0.401. A number of 273 DB compounds were selected based on the efficacy and binding energy thresholds mentioned above, to be considered for future biological testing to identify new potential FAAH inhibitors. Compounds with higher scores are presented in Table 3 based on their decreasing value of the RpS score.

The lead compounds, as seen in Table 3, are either experimental, illicit, or are approved drugs with high toxicity. Therefore, we searched among the 273 DB compounds for approved drugs with a low toxicity profile. We identified five compounds of interest (Table 4).

Binding site analysis of the docked poses was performed for all five selected compounds to assess the possible protein-ligand interactions generating FAAH inhibition.

**Figure 5:**
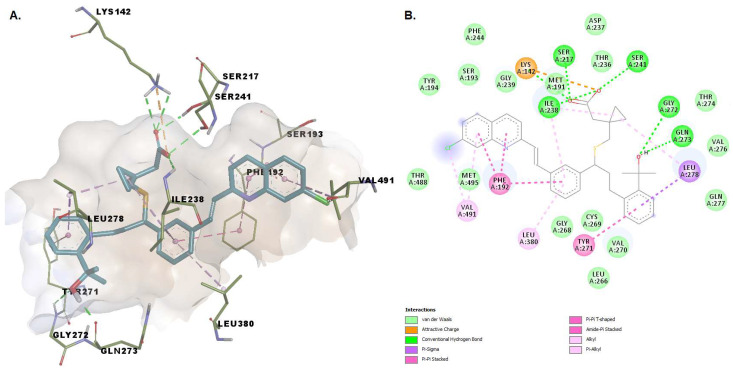
(**A**) 3D binding conformation of montelukast into FAAH binding site; (**B**) 2D diagram of protein-ligand interactions between FAAH and montelukast.

Montelukast seems to be capable of forming multiple conventional hydrogen bonds with the key catalytic residues Ser217, Ser241, Lys142 (Figure 5). Moreover, the charged carboxyl group participates in an attractive interaction with Lys142. The binding conformation is stabilized by pi-pi interactions with Phe192, alkyl, and alkyl-pi interactions with Val491, Ile238, Leu380, and pi-sigma interactions with Leu278, mimicking the conformation the substrate anandamide adopts with the flexible arachidonoyl tail located at the MA/AB interface [20]. The chlorine atom of montelukast is positioned within the MA channel interacting with Val491. The overall goodness of the accomplished pose is confirmed by a favorable (−10.298 kcal/mol) energy of binding and an acceptable ligand efficiency (>0.25).

**Figure 6:**
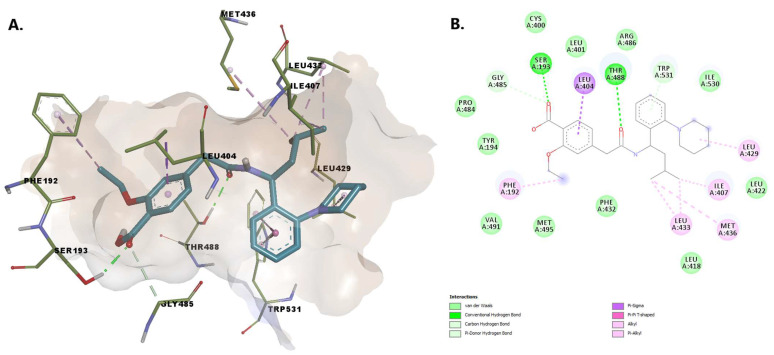
(**A**) 3D binding conformation of repaglinide into FAAH binding site; (**B**) 2D diagram of protein-ligand interactions between FAAH and repaglinide.

The benzoic acid carboxylate moiety of repaglinide makes favorable van der Waals contacts with various hydrophobic residues: Leu401 and Cys400 within the acyl binding pocket, Arg486 located within MA, and Phe432. The oxygens from the carbonylic groups form hydrogen bonds with Ser193 and with Thr488. Alkyl, pi-alkyl, pi-sigma interactions with various residues such as Phe192, Leu433, Met436, Ile407, Leu429, and Leu404 and two carbon-hydrogen bonds (one with Trp531), are established (Figure 6). Residues Phe432, Met495, and Met436 were all shown to be extremely mobile, allowing a change of conformation that leads to a broadened and open membrane access channel with the closure of the acyl chain-binding pocket [41].

**Figure 7:**
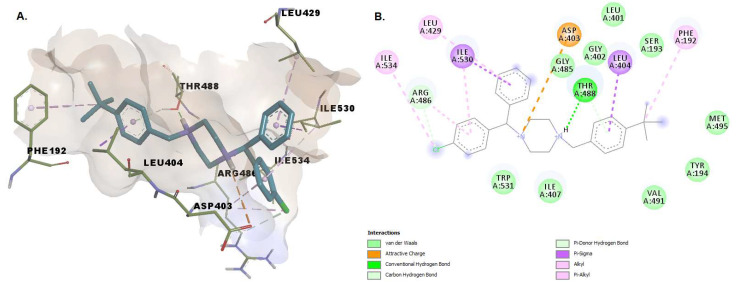
(**A**) 3D binding conformation of buclizine into FAAH binding site; (**B**) 2D diagram of protein-ligand interactions between FAAH and buclizine.

Buclizine has a (4-tert-butylphenyl)methyl moiety embedded in the united section of the MA channel and AB pocket, making stabilizing pi-sigma and pi-alkyl contacts with Leu404 and Phe192, as well as favorable van der Waals contacts with the side chains of a number of hydrophobic residues that line the pocket, including Leu401, Gly485, Gly402, and Met495. The atoms within the piperazine moiety establish hydrogen bonds with Thr488 and van der Waals interactions with Trp531 (involved in substrate orientation during enzymatic hydrolysis). The nitrogen from the piperazine substructure interacts with the charged residues Asp403 (while the phenyl moiety interacts with Arg486) within MA—similar to that of the endogenous fatty acid molecules (Figure 7).

**Figure 8:**
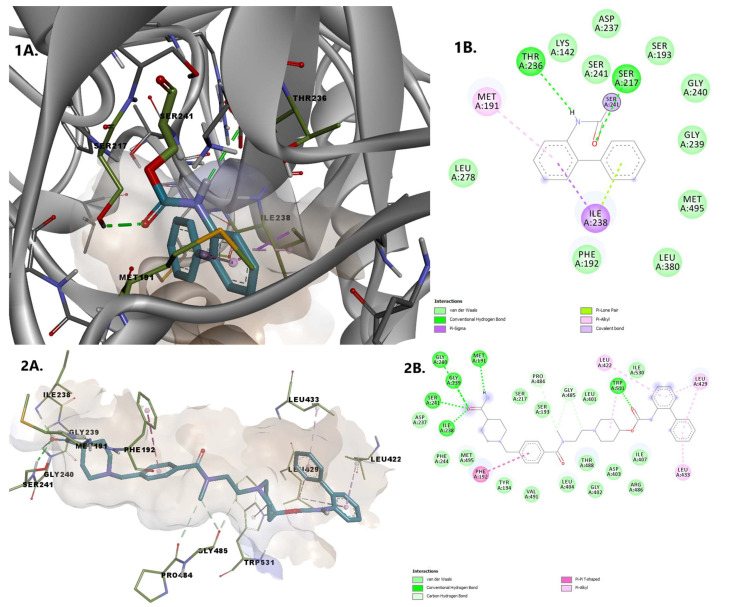
Overlay of revefenacin in the two bound states 1. Covalently bound revefenacin (**A**) 3D binding conformation to FAAH; (**B**) 2D diagram of protein-ligand interactions between FAAH and revefenacin. 2. Non-covalently bound revefenacin (**A**) 3D binding conformation to FAAH; (**B**) 2D diagram of protein-ligand interactions between FAAH and revefenacin.

Noncovalently and covalently bound states of revefenacin were analyzed. Covalent attachment of revefenacin is made through the Ser241 attack on an electrophilic carbonyl group, leading to blockage of the catalytic function of FAAH. The covalent bond is accompanied by various key anchoring interactions such as pi-sigma, pi-alkyl, and van der Walls, established with various residues. The noncovalently bound state of the inhibitor is characterized by important noncovalent interactions stabilizing its binding within the FAAH active site independent of the covalent reaction: conventional hydrogen bonds with Ser241 in the catalytic center as well as with three backbone amide N-H groups of Ile238, Gly239, Gly240, residues defining the oxyanion hole. The oxygen within the carbamoyl group interacts via a hydrogen bond with Trp531. Pi-pi and pi-alkyl interactions are established by the phenyl rings of revefenacin with Phe192 and, respectively, with Leu422, Leu429, Leu433 (Figure 8).

**Figure 9:**
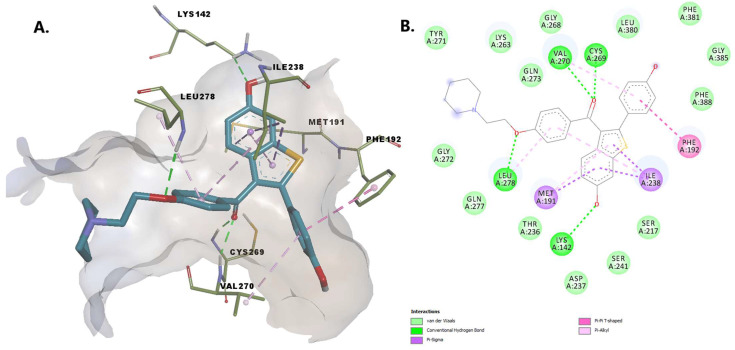
(**A**) 3D binding conformation of raloxifene into FAAH binding site; (**B**) 2D diagram of protein-ligand interactions between FAAH and raloxifene.

Raloxifene establishes hydrogen bonds with Lys142, Leu278, Cys269, and Val270. We consider the interaction between the oxygen from the hydroxy group of the benzothiophene moiety with Lys142 essential, as this deprotonated residue activates the Ser241, allowing the interaction with the amidic head of the substrate during the acylation reaction [20]. The 4-hydroxyphenyl moiety establishes pi-alkyl and pi-pi interactions with residues within the MAC (Val270 and Phe192). Other stabilizing interactions of the protein-ligand complex include pi-sigma interactions with Met191 and Ile238, and van der Waals interactions with other 13 amino acid residues, including Ser217, Ser241 within the catalytic center and Thr236, located in the cytosolic port (Figure 9).

### 2.9. Simulation of FAAH-Montelukast Complex

**Figure 10:**
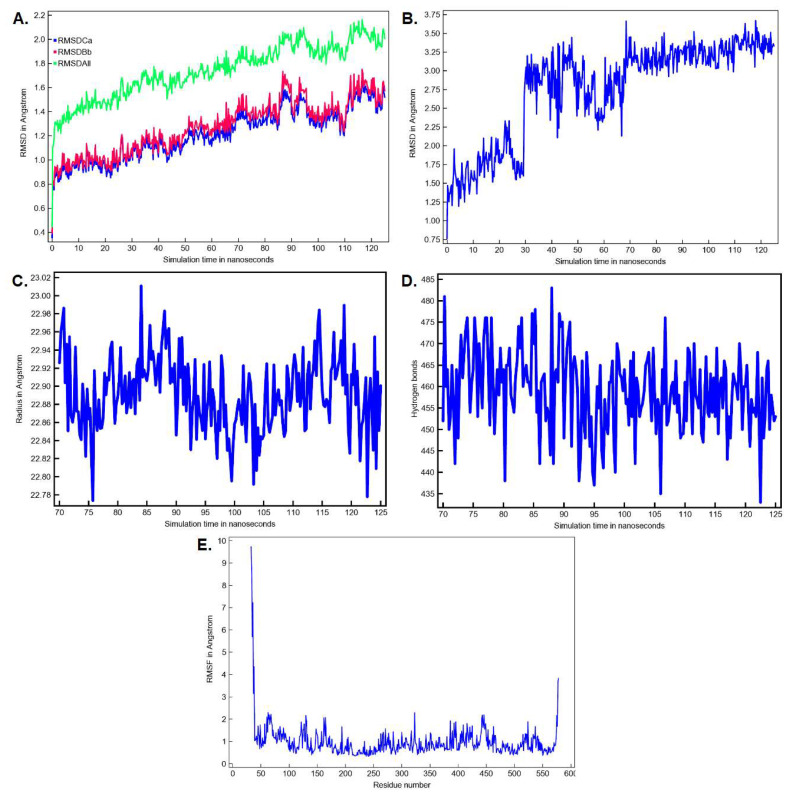
MD simulation parameters for the FAAH-montelukast complex (**A**) The time-dependent RMSD profile of the solute over 125 ns; (**B**) The time-dependent RMSD profile of ligand movement over 125 ns; (**C**) The radius of gyration of the solute over the last 55 ns; (**D**) The number of intramolecular hydrogen bonds over the last 55 ns: (**E**) The RMSF per amino acid residue over the last 55 ns. Ca—C-alpha (central carbon atoms); Bb—backbone atoms; All—all heavy atoms.

**Figure 11:**
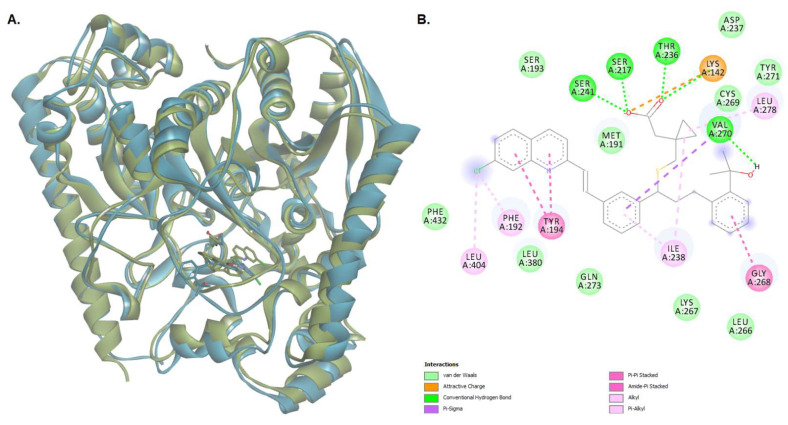
(**A**) The superposition of the protein-ligand complex at the end of the MD simulation over the initial structure (light green—initial conformation; light blue—final conformation); (**B**) 2D diagram of protein-ligand interactions between FAAH and montelukast after the 125 ns MD simulation.

The conformation of the FAAH-montelukast complex resulting from molecular docking analysis was subjected to a 125 ns molecular dynamics (MD) simulation to validate the results and to investigate the complex stability over time. Montelukast was chosen for this experiment as the docking study revealed the formation of several hydrogen bonds between the ligand and all the residues within the catalytic triad. Over the simulation period, the mean RMSD of the protein for all heavy atoms was 1.742 ± 0.218 Å (Figure 10A). The time-dependent evolution of the ligand movement throughout the simulation period is shown in Figure 10B. The ligand movement within the binding pocket seems to reach a certain stability after 70 ns as the RMSD calculated after superposing the complex on its reference structure showed fewer variations. Therefore, further analysis was performed on the last 55 ns of the simulation period. The RMSD of the ligand movement during the last 55 ns ranged from 2.923 to 3.667 Å, with an average of 3.246 ± 0.147 Å, while the mean ligand conformation RMSD was 2.422 ± 0.079 Å. The mean radius of gyration, expressing the compactness of the protein, was 22.892 ± 0.041 Å, ranging from 22.774 to 23.011 Å (Figure 10C). The total number of intramolecular hydrogen bonds varied between 433 and 483, with an average of 249 ± 9.335 (Figure 10D). The fluctuations of amino acid residues during the stimulation are expressed as the root-mean-square fluctuation (RMSF, Å) of each individual residue (Figure 10E). The RMSF plot revealed that the residues within the active site had fewer fluctuations than other residues, indicating that ligand binding hinders residual flexibility. For instance, the RMSF values for the catalytic triad were 0.462 Å for Lys142, 0.345 Å for Ser217 Å, and 0.328 Å for Ser241. The predicted mean free energy of binding of montelukast for the last 55 ns was −62.267 ± 40.546 kcal/mol. The superposition of the FAAH-montelukast complex conformation after 125 ns simulation on the reference structure is shown in Figure 11A. After the 125 ns simulation period, the hydrogen bonds formed between montelukast and Ile238, Gly272, and Gln273 were lost, while new bonds were formed with Val270 and Thr236 (Figure 11B). Moreover, the polar interactions (hydrogen bonds and attractive charges) between the carboxyl moiety and the catalytic triad were not affected throughout the simulation, suggesting that the blockade of the catalytic site is rather stable.

## 3. Discussion

Anandamide is a signaling lipid neurotransmitter regulating various physiological processes [42,43]. Disturbances in its metabolism associated with low anandamide concentrations are reported in various pathologies. The inhibition of FAAH, the key enzyme responsible for the deactivation of this fatty acid, could therefore offer a treatment strategy for several neuropsychiatric and neurological diseases, including chronic pain, inflammation, neurodegeneration, depression, anxiety [11,44,45]. As such, many researchers focused their attention on identifying selective and potent FAAH inhibitors.

In silico studies were undertaken for elucidating the key structural features of such molecules and for understanding the enzyme-substrate interaction. Vacondio et al. developed structure-property relationships to explain the hydrolytic stability of the FAAH-carbamate inhibitor complex [16], while Mor et al. developed 2D-QSAR equations for explaining biphenyl-alkylcarbamates inhibitory activity [17]. 3D-QSAR studies of irreversible inhibitors with the piperazine-carboxamides were performed by Lorca et al. [46].

The first identified reversible inhibitors included: oleoyl-based inhibitors possessing an electrophilic carbonyl, e.g., aldehydes, α-ketoamides, α-ketoesters, and trifluoromethyl ketones [11]. The electrophilic carbon allows the formation of covalent hemiketals with the catalytic nucleophile of the enzyme. Trifluoromethyl ketones were substituted afterward with various monocyclic and bicyclic heterocycles leading to the discovery of ketoheterocycle derivatives of oleic acid as FAAH inhibitors, with the oxazole group emerging as the moiety improving most of the inhibitory potency against this enzyme [19]. The dominant role of the activating heterocycle is its intrinsic electron-withdrawing properties, as demonstrated by assessing the interaction between FAAH and inhibitors such as pyrazole phenylcyclohexylcarbamates derivatives [18], pyridine heterocycles [47], 2,4-dioxopyrimidine-1-carboxamide substituted with a uracil ring [48], etc. Wang and colleagues reported the synthesis and evaluation of benzothiazole-based compounds as potent and selective FAAH inhibitors that lack a structural element such as a-ketone heterocycle, carbamate, or arylurea [49], but with sulfonyl group, the piperidine ring, and the benzothiazole as key components of their activity, as demonstrated by SAR analysis.

However, information on repurposed molecules as potential FAAH inhibitors lacks.

We developed a method for identifying FAAH inhibitors using a combination of ligand-based virtual screening and structure-based drug discovery (graph mining approaches based on inhibitor datasets, SAR, and molecular docking simulation). By employing a multi-step screening protocol, DrugBank compounds were investigated for identifying repurposable molecules.

Based on the results of SAR analysis, a repurposing score was generated and was used as a first screening tool, allowing better identification of potent inhibitors compared to the weak ones. The structure analysis performed identified a profile of the FAAH inhibitors based on the types of cyclic structures and their bonding pattern. Our study focused on this type of compounds because the acyclic FAAH inhibitors generally have low potency. A 3D-QSAR CoMSIA study was previously performed on 90 pyrimidinyl-piperazine-carboxamide derivatives that irreversibly inhibit FAAH and concluded that electrostatic and hydrogen-bond acceptor properties are the most important for the activity of these types of compounds [46]. Similar 3D-QSAR CoMFA and CoMSIA models were constructed for a series of 1,3,4-oxadiazol-2-one derivatives [50]. The importance of the 1,3,4-oxadiazol-2-one scaffold was revealed also in our study by the high P-PR value of fragment PR21. The disadvantage of these studies is their high specificity to a certain type of compounds, while our SAR analysis included most of the various chemical classes of known FAAH inhibitors. A good balance between sensitivity and specificity was obtained for discriminating FAAH inhibitors from decoys, based on the presence of certain Bemis-Murcko skeletons or plain rings. Although artificial intelligence algorithms can typically generate predictive models with high accuracies, the significance of certain structural features for target inhibition is harder to interpret when using such models.

Further filtering was made by using the logistic regression function resulting from the classification analysis and molecular docking scores. Several molecules extracted from DrugBank were predicted as FAAH inhibitors, thus having the potential of being used in the treatment of chronic pain and other disorders. However, most of them are either experimental or investigational, while the approved molecules possess high toxicity and restrained utility, thus making the repurposing of such drugs inappropriate.

After an assessment of the toxicologic profile of the resulted potential inhibitors, five molecules emerged as potentially repurposed FAAH inhibitors: montelukast, raloxifene, repaglinide, revefenacin, and buclizine. The proposed molecules showed common scaffolds with strong FAAH inhibitors (such as piperidine for repaglinide, revefenacin, and raloxifene, piperazine for buclizine) and had high binding affinities for the enzyme. Our molecular docking study indicates that these compounds could similarly interact with FAAH to other known non-covalent inhibitors, and they could achieve potent inhibition of FAAH activity, not by reacting with the nucleophile Ser241, but through shape complementarity to the large substrate-binding pocket in the active site, including the MAC as well as its united section with the acyl binding pocket and through numerous hydrophobic interactions [51]. Apart from buclizine, all the proposed candidates possess hydrogen-bond acceptor atoms as pharmacophores similar to established FAAH inhibitors, forming several hydrogen bonds with key residues [46]. However, similar to many known FAAH inhibitors, revefenacin has a carbamate functional group that can covalently bind the catalytic Ser241, as supported by the docking simulation. Therefore, revefenacin could potentially inhibit FAAH in either a non-covalent or covalent manner. Montelukast, a leukotriene D_4_-receptor antagonist used for asthma treatment, was shown in various preclinical studies to possess analgesic effect in neuropathic pain induced by chronic constriction injury as well as in inflammatory pain [52,53]. Montelukast engaged in interactions with all three catalytic residues and the analysis of the MD simulation revealed that the FAAH-montelukast complex becomes stable after 70 ns, the polar interactions between montelukast and the catalytic triad being preserved after 125 ns. A dual mechanism consisting of leukotriene receptors antagonism and FAAH inhibition could be useful in ameliorating neurodegeneration and other conditions.

Raloxifene is a selective modulator of estrogen receptors, with an estrogen-agonistic effect on bone, and an estrogen-antagonistic effect in the uterus and breast, used for the treatment of osteoporosis in postmenopausal women [54]. Furthermore, observational studies have indicated an analgesic effect of this compound—it was reported to alleviate back and knee pain [55] and to induce marked reduction of skeletal pain in postmenopausal women with osteoporosis and/or osteoarthritis [56], as well as lowering pain in postmenopausal women with fibromyalgia [57]. Our study is offering a potential mechanism of action for this analgesic effect. To the best of our knowledge, there are no similar studies for revefenacin, a new muscarinic M_3_ receptor antagonist used for relieving COPD symptoms [58], repaglinide, an antidiabetic inhibitor of ATP-sensitive potassium channels [59], and buclizine, a sedating antihistamine [60].

Some of the above-mentioned compounds do not or only poorly pass through the blood-brain barrier (BBB). Although a substance passing BBB would exhibit greater analgesic efficacy, it would also possess considerable side effects, e.g., opioids. Nociception is an extremely complex process in which noxious stimuli are detected by peripheral nociceptors, the sensory information is transmitted from the periphery to the central nervous system, where it is analyzed and integrated [61]. The cell bodies of the nociceptors are mainly localized in dorsal root ganglia (DRG) and trigeminal ganglia. The DRG neurons, following nerve injury or inflammation, become an important source of increased nociceptive signaling through increased neuronal excitability and generation of ectopic discharges, mechanisms associated with the development of chronic pain [62]. The DRG and peripheral axons lack an efficient neurovascular barrier [63]. As such, peripherally restricted FAAH inhibitor URB937 presents analgesic effect in various animal models e.g., peripheral nerve injury (chronic sciatic nerve ligation [29], cisplatin-induced neuropathy [64]), migraine (nitroglycerin-induced [65]) arthritis (complete Freund’s adjuvant [66]). In all models, URB937 was as effective or more effective than standard analgesic and anti-inflammatory drugs (indomethacin, gabapentin, dexamethasone) and reversed pain-related responses (mechanical hyperalgesia, thermal hyperalgesia, and mechanical allodynia) in a dose-dependent manner. Therefore, it is proven that a substance might possess a significant analgesic effect even if it does not cross the BBB.

Studies on the distribution of repaglinide and its metabolites in rats indicated rapid and wide distribution to various tissues, including the brain [67]. Montelukast is currently known to cross BBB, its effects being either beneficial or unfavorable, depending on the circumstances [68]. Buclizine passes BBB and has an acceptable safety profile [69]. Despite the low penetrability of the BBB, raloxifene was shown to ameliorate mild cognitive impairments in clinical trials, showed beneficial effects in animal models of Parkinson’s disease and curative effects in animal models of stroke and traumatic brain injury [70]. We proposed the tertiary ammonium derivative (that can be protonated) revefenacin as a candidate since we consider that the information on revefenacin-FAAH interaction offers valuable insight for the researchers in the field. The results retrieved from our in silico screening method warrant future studies to experimentally determine if the proposed molecules can act as FAAH inhibitors and to investigate their therapeutic efficacy in several animal models of neuropathic and inflammatory pain, neurodegeneration, and other ailments.

## 4. Materials and Methods

### 4.1. Datasets Preparation

The chemical structures of human FAAH inhibitors and their corresponding IC_50_ values (M) were acquired from the ChEMBL database (access date: 23 April 2021) using as search target CHEMBL2243 (synonym anandamide amidohydrolase). DataWarrior 5.2.1. software was used to filter the raw dataset and remove the compounds with approximate IC_50_ values. Duplicate records were merged into a single entry using the average IC_50_ value. For all compounds, the IC_50_ values were transformed in the corresponding negative logarithmic values (pIC_50_, M). A decoy set was prepared by searching the ChEMBL database for all the compounds with molecular weight, the logarithm of the partition coefficient, hydrogen bonds donors, and hydrogen bonds acceptors in the same value ranges as the FAAH inhibitors. The results were selected randomly to obtain a decoy set with 10 folds more compounds than the FAAH inhibitors set.

The DrugBank 5.1.8 database of structures was downloaded (access date: 23 April 2021) and used for virtual screening of new potential FAAH inhibitors based on our repurposing strategy.

### 4.2. Molecular Descriptors

A large array of molecular descriptors were calculated based on the SMILES codes obtained from the ChEMBL and their corresponding 3D structures generated with OpenBabel v.2.4.1 [71], using the freely available PaDEL-Descriptor software [72]. The zero variant variables were removed. The ROC analysis was used to choose the cutoff values of each descriptor to have a minimum 0.75 value for sensitivity and specificity. Flag descriptors (flg) were computed to indicate if the value of the corresponding descriptor is above or under the defined cutoff value using the following formula.

(2) $$\mathrm{flg}-\mathrm{descriptor}=\{\begin{array}{c} 0,\;\;\mathrm{descriptor}<\mathrm{cutoff}\\ 1,\;\;\mathrm{descriptor}\geq \mathrm{cutoff} \end{array}$$

The flag values were used to perform a binary logistic regression to discriminate the FI-S compounds from those in the FI-W set. The obtained equation was used to select new potential FAAH inhibitors from the DB set.

### 4.3. Bemis-Murcko Skeletons and Murcko Frameworks Analysis

Both sets of compounds FI and DCY, as well as DB, were processed using DataWarrior 5.2.1 to generate the Murcko frameworks (MF) and Bemis-Murcko (BM) skeletons representing the structural molecular frameworks incorporating only the rings and the chains connecting them. Each BM skeleton was analyzed based on its frequency of occurrence. The significance of the differences in the occurrence frequency for each scaffold in active and inactive sets was determined using a non-parametric chi-square test (*p* < 0.05).

### 4.4. Plain Ring Analysis

DataWarrior 5.2.1 software was used to generate all ring systems existing in each compound from FI and DCY sets as well as from DB. Each structure was divided into multiple fragments based on each cyclic structure. The single bonded substituents were erased, keeping only the double-bonded heteroatoms connected directly to the ring system.

### 4.5. Molecular Docking

A molecular docking study was employed to establish a relationship between predicted and experimental binding parameters for FAAH inhibitors and for identifying novel potential inhibitors among the repurposable candidates.

The crystal structure of a humanized variant of rat FAAH (h/r FAAH) bound to a covalent inhibitor in a noncovalent intermediate binding state (PDB ID: 3PPM) was retrieved from the RCSB PDB database (access date: 13 September 2021). Although there are many solved crystal structures of FAAH bound to both covalent and noncovalent inhibitors, we chose the protein with the highest resolution (1.78 Å), which is also the only humanized variant bound in a noncovalent manner to an inhibitor.

FAAH protein structure was prepared for docking using YASARA Structure [73]. The protein was cleaned by correcting structural errors, was protonated according to the physiological pH (7.4) and the hydrogen-bonding network was optimized. Moreover, the bound inhibitor (JG1), 1-dodecanol, di(hydroxyethyl)ether, chloride, and fluoride ions and water molecules were removed from the complex, and only chain A was kept for the docking experiments.

The 3D structures of FI and DB sets were prepared for docking by energy minimization with Open Babel, using MMFF94s forcefield and 1500 steps with the steepest descent algorithm. Ligand structures were also protonated at pH 7.4 and were docked using AutoDock Vina v1.1.2 algorithm within YASARA. The docking searching space was set to include the FAAH catalytic triad (Ser217, Sert241, and Lys142). The grid box (33.38 × 33.38 × 33.38 Å) included the binding sites of both covalent and noncovalent inhibitors and was selected by superposing several crystal structures of FAAH in complex with noncovalent inhibitors (PDB IDs: 3QJ9 [74], 4DO3 [75], 6MRG [76]) on 3PPM [51]. A total of 12 docking runs were performed for each ligand, with the exhaustiveness parameter set to 25. Docking results were returned as the binding energy (ΔG, kcal/mol) and ligand efficiency (ΔG\no. of heavy atoms) of the best binding pose for each ligand.

The conformations of the predicted protein-ligand complexes and molecular interactions were analyzed using BIOVIA Discovery Studio Visualizer (BIOVIA, Discovery Studio Visualizer, Version 17.2.0, Dassault Systèmes, 2016, San Diego, CA, USA). The molecular docking protocol was validated by docking the co-crystallized FAAH inhibitor JG1 to the target protein and calculating the root-mean-square deviation (RMSD, Å) between the experimental and predicted complexes after superposition.

The binding poses of repurposable candidates with the best results were further refined using YASARA, by redocking with AutoDock Vina local search algorithm and energy minimization of the complex with NOVA forcefield. Moreover, if we suspected a hit compound of potential covalent interaction with the catalytic Ser241, the ligand was redocked using the AutoDock Vina covalent docking procedure.

### 4.6. Molecular Dynamics Simulation

A molecular dynamics simulation was performed to further validate the docking results and analyze the protein-ligand complex stability. Based on the repurposing screening results, the docking pose of one promising candidate for potential FAAH inhibition was selected for the simulation. The simulation of the chosen complex was performed with YASARA Structure. Firstly, the hydrogen bonding network was optimized to increase stability, while the protonation states were fine-tuned at the physiological pH (7.4) [77]. The simulation system was neutralized by adding NaCl ions at 0.9% concentration. Clashes were removed by performing steepest descent and simulated annealing minimizations. The simulation duration of the predicted complex was 125 ns. AMBER14 force field was used for the protein [78], GAFF2 [79] and AM1BCC [80] for ligand and TIP3P for water. The cut-off for van der Waals forces was 8 Å [81], while the electrostatic forces were treated using the Particle Mesh Ewald algorithm and no cutoff was applied [82]. The integration of motions equations was performed with a multiple timestep of 2.5 fs for bonded and 5 fs for non-bonded interactions at 298 K and 1 atm (isothermal-isobaric ensemble) [83]. The free energy of binding (kcal/mol) of the simulated ligand was estimated using the Poisson-Boltzmann (MM/PBSA) method, excluding the entropic term.

### 4.7. Statistical Analysis

ROC curve analysis and statistical tests (independent sample *t*-test, Mann-Whitney) were performed with GraphPad Prism v.9.1.0 (GraphPad Software Inc., San Diego, CA, USA) and IBM SPSS Statistics 24–26 (Armonk, New York, NY, USA). Data distribution was established using D’Agostino & Pearson test. The statistical significance threshold for all tests was α = 0.05.

## 5. Conclusions

We implemented a step-by-step screening algorithm for identifying new potential FAAH inhibitors within DrugBank database. The interactions of the prioritized hits, the approved drugs montelukast, repaglinide, revefenacin, raloxifene and buclizine, were confirmed by docking with the molecular target. The screening results were further supported by MD simulation for montelukast. Our results indicate these compounds could potentially be repurposed for treating chronic pain through FAAH pharmacological inhibition. The study revealed also the major geometric requirements for a FAAH inhibitor highlighting the importance of particular heterocyclic rings and their binding patterns.

## Figures and Tables

**Table 1:** The range of the number of major substitution groups for the structures included in the two sets (FI, DCY).

Set	nF	nCl	nBr	nI	nX	nOH	nCOOH	nCONH_2_	nNH_2_	nNO_2_
FI	0–8	0–3	0–2	0–1	0–8	0–2	0–1	0–1	0–1	0–2
DCY	0–10	0–6	0–5	0–3	0–10	0–4	0–3	0–1	0–3	0–2

**Table 2:** List of molecular descriptors with significantly different values in relation to strong and weak FAAH inhibitors. The cutoff value indicates the threshold for discriminating strong FAAH inhibitors.

Code	Class	Type	Cutoff	Descriptor’s Mathematical Representation
CrippenLogP	2D	Crippen	>4.582	Crippen’s LogP
SpMAD_D	2D	Topological	>12.569	spectral mean absolute deviation from the topological distance matrix
SpMax5_Bhi	2D	Burden modified	>3.333	largest absolute eigenvalue of Burden modified matrix—n 5/weighted by relative first ionization potential
Au	3D	WHIM	>98.038	a total size index—unweighted
Ae	3D	WHIM	>96.826	a total size index—weighted by relative Sanderson electronegativities
Ai	3D	WHIM	>100.252	a total size index- weighted by relative first ionization potential
As	3D	WHIM	>97.668	a total size index—weighted by relative I-state
Av	3D	WHIM	>81.277	a total size index—weighted by relative van der Waals volumes
RDF85m	3D	RDF	>5.699	radial distribution function—085/weighted by relative mass
WPSA-1	3D	CPSA	>382.111	partial positive surface area (PPSA-1) × total molecular surface area/1000
WPSA-2	3D	CPSA	>767.505	partial positive surface area × total positive charge on the molecule (PPSA-2) × total molecular surface area/1000

**Table 3:** The top 20 candidates for repurposing studies based on their RpS score values.

Code	Name	RpS	Category
DB06442	Avasimibe	15.38	investigational
DB08078	{4-[3-(4-acetyl-3-hydroxy-2-propylphenoxy)propoxy]phenoxy}acetic acid	15.38	experimental
DB12390	MBX-8025	15.38	investigational
DB07142	5-[(3R)-3-(5-methoxy-3′,5′-dimethylbiphenyl-3-yl)but-1-yn-1-yl]-6-methylpyrimidine-2,4-diamine	14.19	experimental
DB07144	5-[(3R)-3-(5-methoxy-2′,6′-dimethylbiphenyl-3-yl)but-1-yn-1-yl]-6-methylpyrimidine-2,4-diamine	14.19	experimental
DB11718	Encorafenib	13.82	approved
DB12170	Veledimex	13.32	investigational
DB12226	Terameprocol	13.32	investigational
DB08896	Regorafenib	13.25	approved
DB12524	BI-671800	13.22	investigational
DB09171	β-Methylfentanyl	13.10	illicit
DB09174	Lofentanil	13.10	illicit
DB09179	R-30490	13.10	experimental
DB13016	LY-2300559	13.02	investigational
DB15052	Ansofaxine	13.02	investigational
DB13232	Suxibuzone	11.33	experimental
DB04741	Myxothiazol	8.61	experimental
DB01347	Saprisartan	7.54	experimental
DB07238	Nesbuvir	7.52	investigational
DB16169	Fonadelpar	7.51	investigational

**Table 4:** Top potential repurposing candidates based on the RpS and molecular docking score values and on the toxicological profile.

Code	Name	RpS	ΔG (kcal/mol)	LE	No. of Contacts	H-Bonding Residues	H-Bond Length (Å)	Category
DB00481	Raloxifene	7.23	−10.464	0.3078	21	Lys142	2.51	approved
						Cys269	1.84	investigational
						Val270	2.28	
						Leu278	2.57	
DB00471	Montelukast	7.06	−10.298	0.2512	27	Lys142	2.69	approved
						Lys142	2.53	
						Ser217	2.17	
						Ser241	2.80	
						Gly272	2.55	
						Gln273	1.94	
DB11855	Revefenacin	6.91	−12.020	0.2732	27	Ile238	1.81	approved
	(noncovalent)					Gly239	2.60	investigational
						Gly240	3.00	
						Sert241	2.33	
						Trp531	1.85	
						Met191	2.65	
	Revefenacin		−5.710	0.3807	14	Ser217	2.16	
	(covalent)					Thr236	2.72	
DB00912	Repaglinide	6.89	−9.306	0.2820	21	Ser193	2.04	approved
						Thr488	2.13	investigational
DB00354	Buclizine	6.87	−9.808	0.3164	21	Thr488	1.70	approved

## Data Availability

Publicly available datasets were analyzed in this study. This data can be found here: https://www.ebi.ac.uk/chembl/ and https://go.drugbank.com/ (access date: 23 April 2021).
